# L-amino acid oxidase from *Bothrops atrox* snake venom triggers autophagy, apoptosis and necrosis in normal human keratinocytes

**DOI:** 10.1038/s41598-018-37435-4

**Published:** 2019-01-28

**Authors:** Fernanda Costal-Oliveira, Stephanie Stransky, Clara Guerra-Duarte, Dayane L. Naves de Souza, Dan E. Vivas-Ruiz, Armando Yarlequé, Eladio Flores Sanchez, Carlos Chávez-Olórtegui, Vania M. M. Braga

**Affiliations:** 1 Departamento de Bioquímica-Imunologia, Instituto de Ciências Biológicas, Universidade Federal de Minas Gerais, Belo Horizonte, 31270-901 Minas Gerais Brazil; 2 Centro de Pesquisa e Desenvolvimento, Fundação Ezequiel Dias, 30510-0103 Belo Horizonte, Minas Gerais Brazil; 3 Laboratorio de Biología Molecular, Facultad de Ciencias Biológicas, Universidad Nacional Mayor de San Marcos, Lima, Peru; 4 Cardio-Respiratory Section, National Heart and Lung Institute, Faculty of Medicine, Imperial College London, Sir Alexander Fleming Building, SW7 2AZ London, UK

## Abstract

Snake venom L-amino acid oxidases (LAAOs) are flavoproteins, which perform diverse biological activities in the victim such as edema, myotoxicity and cytotoxicity, contributing to the development of clinical symptoms of envenomation. LAAO cytotoxicity has been described, but the temporal cascade of events leading to cell death has not been explored so far. This study evaluates the involvement of LAAO in dermonecrosis in mice and its cytotoxic effects in normal human keratinocytes, the major cell type in the epidermis, a tissue that undergoes extensive necrosis at the snakebite site. Pharmacological inhibition by the antioxidant NAC (N-acetyl cysteine) prevented *B*. *atrox* venom-induced necrosis. Consistent with the potential role of oxidative stress in wounding, treatment with purified LAAO decreased keratinocyte viability with an Effective Concentration (EC_50_) of 5.1 μg/mL. Cytotoxicity caused by LAAO was mediated by H_2_O_2_ and treated cells underwent autophagy, followed by apoptosis and necrosis. LAAO induced morphological alterations that precede cell death. Our results show the chronological events leading to cell death and the temporal resolution from autophagy, apoptosis and necrosis as distinct mechanisms triggered by LAAO. Fluorescently-labelled LAAO was efficiently and rapidly internalized by keratinocytes, suggesting that catalysis of intracellular substrates may contribute to LAAO toxicity. A better understanding of LAAO cytotoxicity and its mechanism of action will help to identify potential therapeutic strategies to ameliorate localized snake envenomation symptoms.

## Introduction

Snakebites constitute a public health problem worldwide and are considered a priority neglected tropical disease by the World Health Organization^1^. Accidents caused by snakes are a major occupational health issue in rural areas and result in a high human morbidity and mortality in tropical countries^2^. *Bothrops atrox* snakes (Viperidae: Crotalinae), the common Lancehead, are responsible for the great majority of envenomation accidents in rainforests in South America, and is the leading cause of human fatalities provoked by snakes in this area^3^. Bothropic envenomation is characterized by serious life threatening, local and systemic effects, including coagulopathies, acute renal failure, cardiotoxicity, spontaneous bleeding and bruises^3–8^. Local bleeding, edema, pain, redness and hemorrhagic blisters can be observed, and necrosis at the bite site can lead to extensive scarring and amputation of the affected limb^6,7^. Although the role of metalloproteinases and phospholipases A_2_ in these local pathological symptoms are well characterized^9–11^, the involvement of other proteins, such as L-amino acid oxidase has not been established so far.

L-amino acid oxidases (LAAO - EC 1.4.3.2) are flavoproteins found in a wide range of organisms, invertebrates and vertebrates, as bacteria, fungus, fish and in snake venoms^12–14^. LAAOs catalyze the stereospecific oxidative deamination of L-amino acids to produce the corresponding α-keto acids, hydrogen peroxide (H_2_0_2_) and ammonia^15^. Snake venom-LAAOs (SV-LAAOs) exhibit substrate specificity for hydrophobic or aromatic amino acids^16–18^. Although LAAO is not amongst the most abundant and studied toxins, this protein is prevalent in many snake venoms^19^. In mammalian species, LAAOs may be a housekeeping protein that together with D-amino acid oxidases are involved in amino acid metabolism, neuromodulation and in the innate immune defense^20,21^. The precise role of SV-LAAOs in the context of venom toxicity and its consequences to the prey are not very clear.

The percentage of LAAO in snake venoms can vary from 0,15% (*Naja naja oxiana*) to 25% (*Bungarus caeruleus*)^22–24^. In *B*. *atrox* venom, LAAO content was previously determined as 10.5% of the total proteins^25^. SV-LAAOs are involved in edema, hemolysis and myotoxicity, which may contribute for the development of envenomation symptoms^16,18,26–28^. A high correlation between *in vitro* LAAO activity and *in vivo* necrosis was reported in the bothropic venom, which suggests LAAO involvement in the dermonecrosis caused by the venom^29^. Cellular toxicity induced by SV-LAAOs has been shown in mammalian tumor cell lines^14,17,30^ and primary cells such as neutrophils^31^. However, dissection of LAAO effects in normal epithelial cells and the temporal distribution of cell death mechanisms triggered by this protein are poorly understood.

In this work, we evaluated distinct mechanisms of cell death triggered by exposure of keratinocytes, the main cell type in the epidermis, to LAAO. Cell death mechanisms (*i*.*e*. autophagy, apoptosis or necrosis) are classified according to morphological structure, enzymological criteria, functional aspects or immunological characteristics. However, interplay between these distinct pathways is often observed^32^. Autophagy also plays a crucial role in the adaptive response, providing nutrients and energy to assist during stress conditions such as starvation^33^. Autophagy has been previously reported as a cellular response against snake toxins and, in some cases, to contribute to apoptotic cell death^34,35^.

Cell death by apoptosis or necrosis has been reported in cells treated with specific snake toxins such as lectin, LAAO and metalloproteinases^17,36–38^. Apoptotic cells show morphological alterations such as chromatin condensation, cell shrinkage, nuclear fragmentation and formation of plasma-membrane blebs. In apoptosis, membrane integrity is maintained until late in the process^39^. On the other hand, necrotic cell death is characterized by an increase in cell volume and organelle swelling, which result in plasmatic membrane rupture and extravasation of internal content^32,40^. Organelle membrane damage releases proteolytic enzymes from lysosomes and consequently lead to cell destruction^39^.

In the present work, we purified L-amino acid oxidase from *B*. *atrox* venom and determined its biochemical properties, cytotoxic effects and mechanism of action in primary keratinocytes, as the epidermis is a tissue affected by local envenomation. Our results showed that LAAO is cytotoxic to human keratinocytes, as it decreased cell viability and induced morphological alterations and cell death by three different pathways: autophagy, necrosis and apoptosis. Our data contribute to a better understanding of the mechanisms of action of LAAO at the cellular level and provide insights into its contribution to localized tissue necrosis during envenomation. By establishing the molecular mechanisms that underlie the deleterious effects triggered by LAAO and other venom toxins, we are able to design strategies to counteract the local symptoms that are currently poorly neutralized by antivenom.

## Results

### Evidence of LAAO involvement in tissue injury

**Figure 1:**
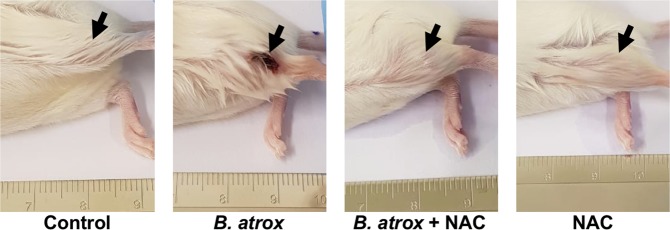
Dermonecrosis inhibition by NAC. Mice gastrocnemius muscle was injected with 100 μL of PBS (negative control), *B*. *atrox* venom (150 µg/animal), *B*. *atrox* venom (150 µg/animal) + NAC (1:50 w/w) or NAC. After 72 h animals were euthanized and the tissue injury was evaluated.

We have first investigated the involvement of LAAO in the *in vivo* symptoms of *B*. *atrox* envenomation. LAAO contribution for the local tissue injury was assessed by *in vivo* assay using N-acetyl cysteine (NAC), an antioxidant and LAAO inhibitor^41,42^. Swiss mice were injected in the gastrocnemius muscle with 100 μL of (i) Phosphate Buffered Saline (PBS), used as control, (ii) *B*. *atrox* venom, (iii) *B*. *atrox* venom + NAC (1:50 w/w) or (iv) NAC (Fig. 1). Mice injected with *B*. *atrox* venom presented dermonecrosis, in addition to hemorrhage (Supplementary Fig. S1) whilst animals that received *B*. *atrox* venom + NAC did not present any of these symptoms. These data indicate that blocking oxidative stress strongly prevented the wounding caused at the venom injected site, suggesting that LAAO’s contribution to local oxidative events may underlie tissue injury caused by *B*. *atrox* venom.

### LAAO purification and characterization

**Figure 2:**
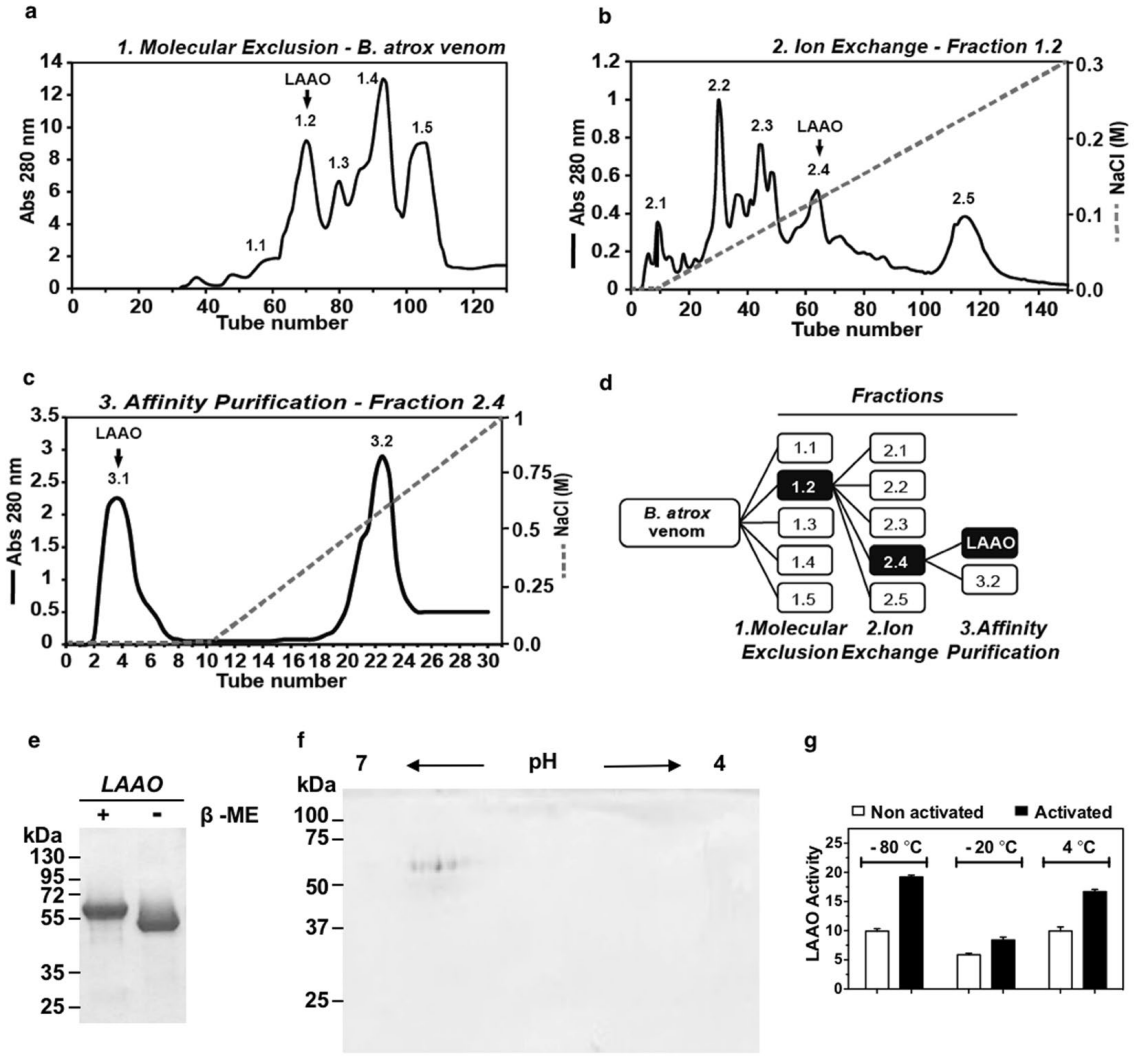
Isolation and characterization of L-amino acid oxidase from *B*. *atrox*. *B*. *atrox* venom was fractionated using **(a)** Molecular exclusion in Sephadex S-200 and **(b)** Ion exchange chromatography in DEAE-Sepharose. **(c)** The enzymatic active fraction was selected and applied to a HiTrap Heparin Hp in HPLC Shimadzu. In all chromatographic steps fractions were monitored at 280 nm. **(d)** Schematic diagram of LAAO purification. **(e)** LAAO with (+) and without (−) β-mercaptoethanol (β-met) was applied to a 12% polyacrylamide gel and protein was stained with Coomassie Blue (cropped image). Full-length gel is presented in Supplementary Fig. S2. (**f**) For 2D electrophoresis, LAAO (30 μg) was applied to a pH 4–7 IPG strip and electrophoresis was carried out on 12% acrylamide gel. **(g)** Temperature and activation effects on LAAO activity. LAAO aliquots were kept at −80 °C, −20 °C or 4 °C for 5 days. Their activity was tested with (black bars) or without (white bars) pre-activation with acetate buffer pH 5.0 for 30 min at 37 °C. Absorbance was determined at 490 nm and LAAO activity was expressed as ΔA492 nm/min/mg).

LAAO was purified and its cellular effects on keratinocytes were further studied. For the purification process, three chromatographic steps were performed, (1) molecular exclusion, (2) ion exchange and (3) affinity purification as described by Ciscotto *et al*.^26^. In the first step, *B*. *atrox* venom was fractionated using Sephacryl S-200 column in five main fractions, of which LAAO activity was found in the second fraction (Fraction 1.2; Fig. 2a). Fraction 1.2 was concentrated and subsequently applied to the ion exchange DEAE Sepharose CL 6B column. Five main fractions were separated and LAAO activity was detected in Fraction 2.4 (Fig. 2b). This fraction was concentrated and placed in a HiTrap Heparin Hp column, from which purified LAAO was released in the void volume (Fraction 3.1; Fig. 2c). The chromatographic process is summarized on Fig. 2d and Supplementary Table S1. One dimensional SDS-PAGE separation of LAAO revealed a single band under reducing or no reducing conditions (Fig. 2e; see Supplementary Fig. S2 for full-length gel). Two-dimensional electrophoresis demonstrated the presence of 9 protein spots with isoelectric points ranging from 5.9 to 6.5 (Fig. 2f). Protein purity and molecular mass of 57 kDa were confirmed by mass spectrometry (Supplementary Fig. S3).

In order to assess LAAO stability, the enzymatic activity was evaluated after keeping LAAO at −80 °C, −20 °C or 4 °C for 5 days, with or without LAAO reactivation using sodium acetate buffer pH 5.0 for 30 min at 37 °C (Fig. 2g). LAAO was more stable when kept at −80 °C, followed by 4 °C. For all temperatures tested, an increased LAAO enzymatic activity was observed after reactivation treatment. For all subsequent assays in this study, LAAO was kept at −80 °C and reactivated immediately before experiments.

### LAAO triggers H_2_O_2_ mediated cytotoxicity and affects cells morphology

**Figure 3:**
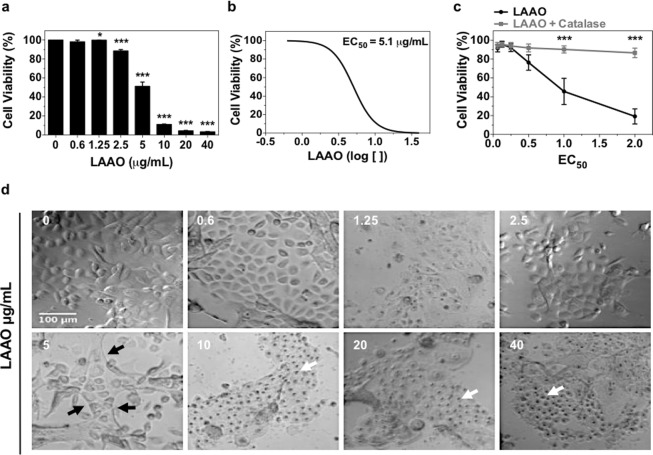
LAAO cytotoxicity against keratinocytes. (**a)** Concentration-response of LAAO cytotoxicity. Cells were incubated with different concentration of LAAO (0.6–40 μg/mL) and after 24 hours cell viability was analyzed using Alamar Blue reagent. **(b)** EC_50_ curve (concentration capable of reducing cell viability to 50%) using Graph Pad Prism software. **(c)** Catalase effect (100 μg/mL) in the cytotoxicity trigged by LAAO (0, 0.25, 0.5, 1 and 2EC_50_). Cells were incubated with LAAO, with or without catalase and after 24 hours cell viability was analyzed. Values represent means of three independent assays and error bars indicate standard error of the means (SEM). **(d)** Morphological alteration in LAAO treated keratinocytes. Following incubation with different LAAO concentrations, cells were fixed and phase contrast images were acquired. Black arrows indicate colony edges retractions and white arrows show pyknotic nuclei. *p ≤ 0.05, ***p ≤ 0.001. Scale bar = 100 μm.

We next optimized a model to investigate the cellular mechanisms of cell death caused by LAAO treatment. Cells were incubated with different concentrations of LAAO (0.6–40 µg/mL) and a concentration-dependent decrease in cell viability was observed (Fig. 3a). The concentration that reduced cell viability to 50% (Effective Concentration - EC_50_) was calculated as 5.1 µg/mL (Fig. 3b). In accordance with previous work^14^, incubation with purified LAAO and catalase (100 µg/mL), an H_2_O_2_ scavenger, maintained cell viability around 80–90% for concentrations of 1EC_50_ and 2EC_50_ (p ≤ 0.001). These data suggest that H_2_O_2_ has an important role in LAAO cytotoxicity (Fig. 3c).

**Figure 4:**
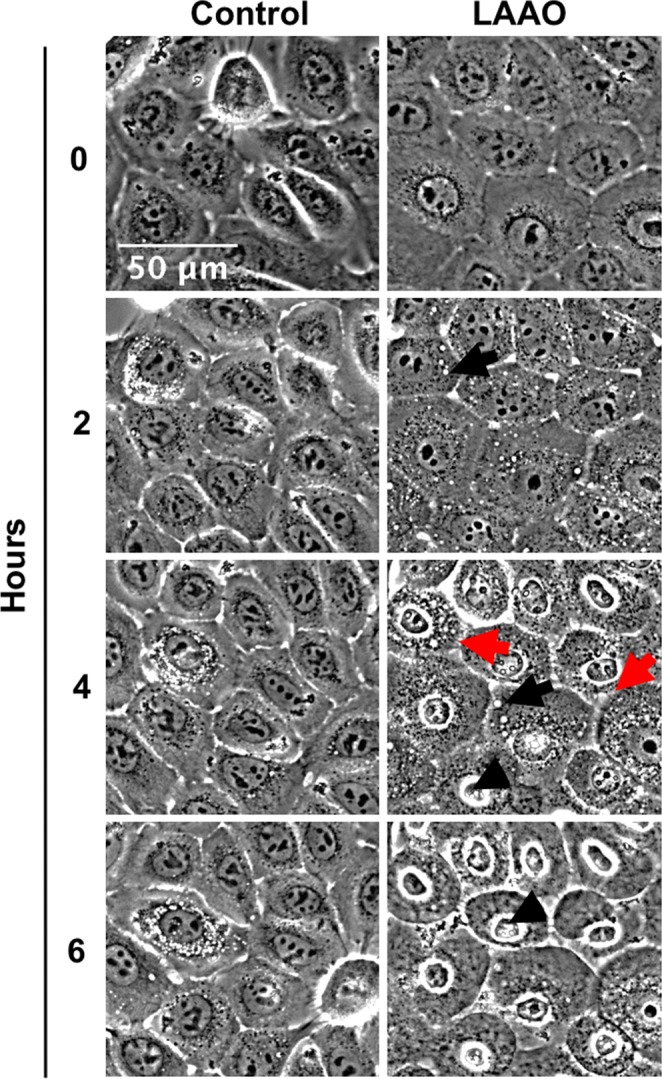
Morphological analysis of cells treated with LAAO. Keratinocytes were treated with LAAO (2EC_50_) and imaged using phase contrast microscopy for 6 hours. Non-treated cells were used as control. Black arrows: cytoplasmic vesicles; red arrows: cell retraction; black arrows head: pyknotic nuclei. Images were acquired in a widefield timelapse microscopy. Scale bar = 50 μm.

Cell morphology was analyzed after 24 hours treatment with different LAAO concentrations (0.625–40 µg/mL) (Fig. 3d). Keratinocytes treated with 5 µg/mL of LAAO showed retraction of the colony border (black arrows). Nuclei with condensed chromatin (pyknotic) (white arrows) were observed at concentrations higher than 10 µg/mL, suggesting that apoptotic cell death may have been triggered. Next, cells treated with 2EC_50_ were evaluated regarding its morphology using video time-lapse microscopy during 6 hours (Fig. 4 and Supplementary Video S1 and S2). After 2 hours of LAAO treatment, enlarged vesicles were observed in the cytoplasm (black arrows). After 4 hours, detachment between cells (red arrows) and pyknotic nuclei (black head arrows) were also detected.

### LAAO triggers autophagy followed by apoptosis and necrosis

The morphological characteristics observed in the phase contrast images and video time-lapse are suggestive of apoptotic events. For completeness, we also evaluated autophagy and necrosis.

**Figure 5:**
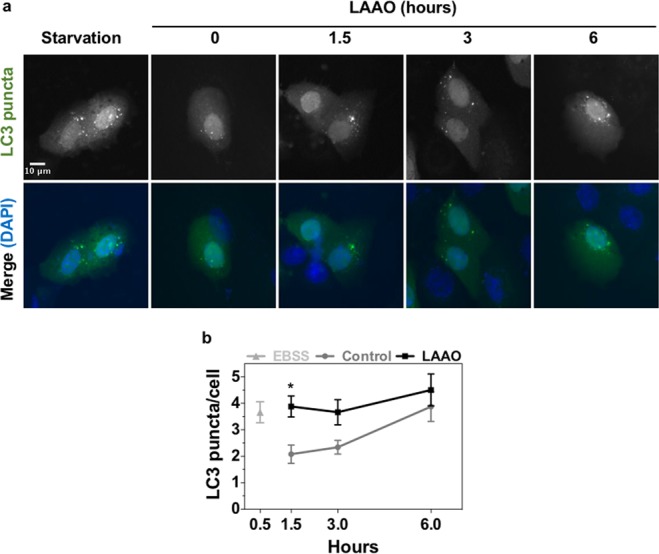
LAAO triggers autophagy in keratinocytes. (**a**) Cells transfected with LC3-GFP and treated with 2EC_50_ of LAAO for 1.5; 3 e 6 hours. Non-treated cells were used as negative control and starved cells (treated with EBSS for 30 min) were considered positive control. Cells were incubated with DAPI for nuclei staining and images were acquired on a fluorescence microscope. Scale bar = 10 μm. **(b)** Average number of LC3 puncta per transfected cell. Values represent means of three independent assays and error bars indicate standard error of the means (SEM). *p ≤ 0.05.

Autophagic process is characterized by the association of Microtubule associated protein 1 light chain 3 (LC3) to autophagosomes. Levels of autophagic responses can be determined as LC3 puncta using GFP-LC3 transfected into keratinocytes^43^. Figure 5a shows representative cells of each tested condition. The number of LC3 puncta per cell was quantified and normalized to control (arbitrarily set as 1) (Fig. 5b). After 1.5 hour incubation with LAAO, a two-fold increase in the levels of LC3 puncta was detected relative to control samples (p ≤ 0.05). No significant difference was observed for later time points.

**Figure 6:**
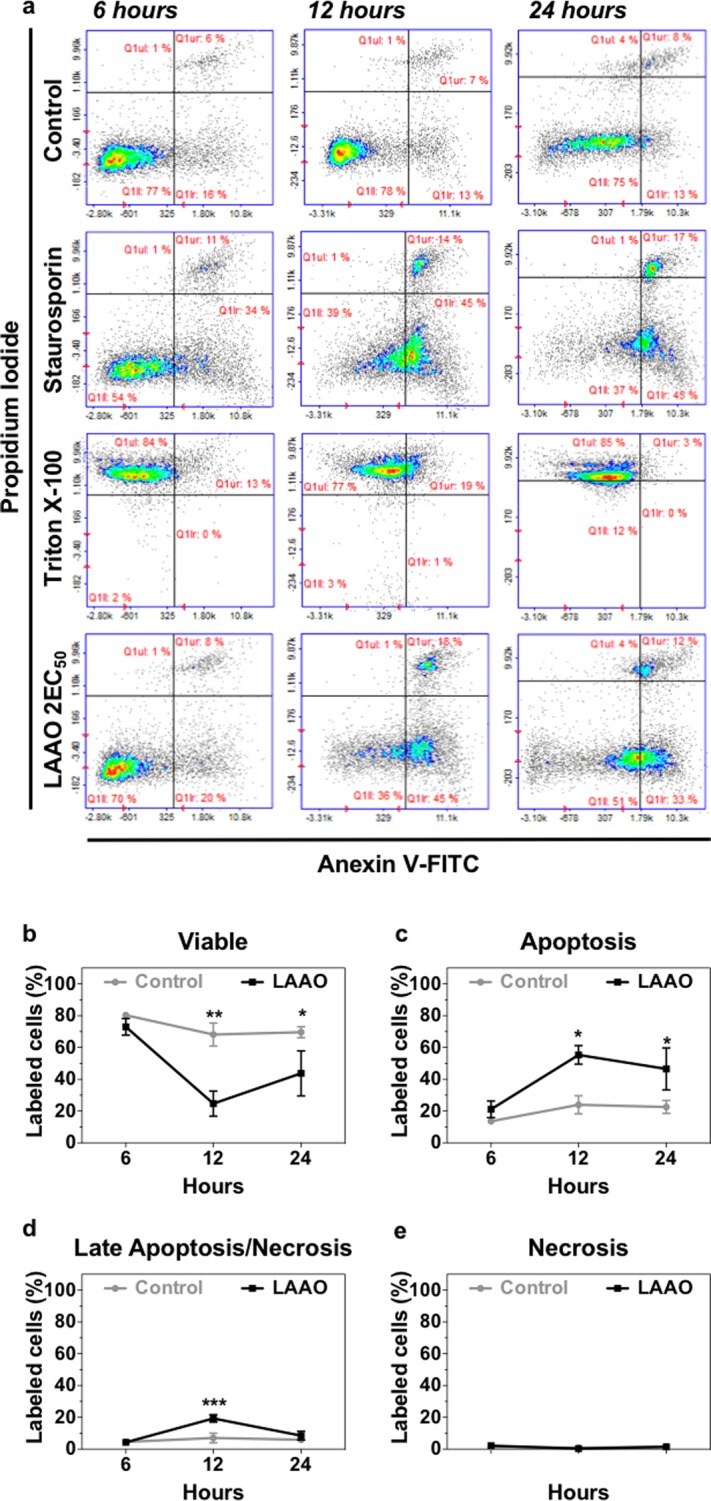
Apoptosis pathway (6, 12 and 24 hours) evaluation after LAAO treatment. Keratinocytes were treated with LAAO (2EC_50_) for different time points and results were obtained using flow cytometry. **(a)** Dot plots showing cells labeled with Annexin-V and/or PI. Non-treated cells were used as negative control and cells treated with 1 μM Staurosporine or Triton X-100 0,1% were used as positive control for apoptosis and necrosis, respectively. **(b**–**e)** Graphs show quantifications of the proportion of **(b)** Viable cells. **(c)** Apoptotic cells (labelled with Annexin-V). **(d)** Late apoptotic/necrotic cells (labelled with Annexin-V and Propidium Iodide (PI)). **(e)** Necrotic cells (labelled with PI). Values represent means of three independent assays and error bars indicate standard error of the means (SEM). *p ≤ 0.05, **p ≤ 0.01, ***p ≤ 0.001.

Cells were treated with LAAO at 2EC_50_ for 6, 12 and 24 hours and stained with Annexin-V and PI to assess levels of apoptosis and necrosis (Fig. 6) respectively, and the percentage of labeled cells was quantified (Fig. 6b–e). After 6 hours treatment, no significant difference was detected between control and LAAO-treated samples. Incubation with purified LAAO significantly decreased cell viability after 12 (p ≤ 0.01) and 24 hours treatment (p ≤ 0.05) (Fig. 6b). A significant increase in the percentage of apoptotic cells was observed after 12 and 24 hours incubation (24% of apoptotic cells in control and 45–55% in treated cells) (p ≤ 0.05) (Fig. 6c). Furthermore, a transient increase in late apoptosis or necrosis was seen after 12 hours post-treatment (p ≤ 0.001) (Fig. 6d). However, there was no significant difference in the level of necrotic cell between control and treated samples (cells labeled only with PI) in any of the time points tested (Fig. 6e).

**Figure 7:**
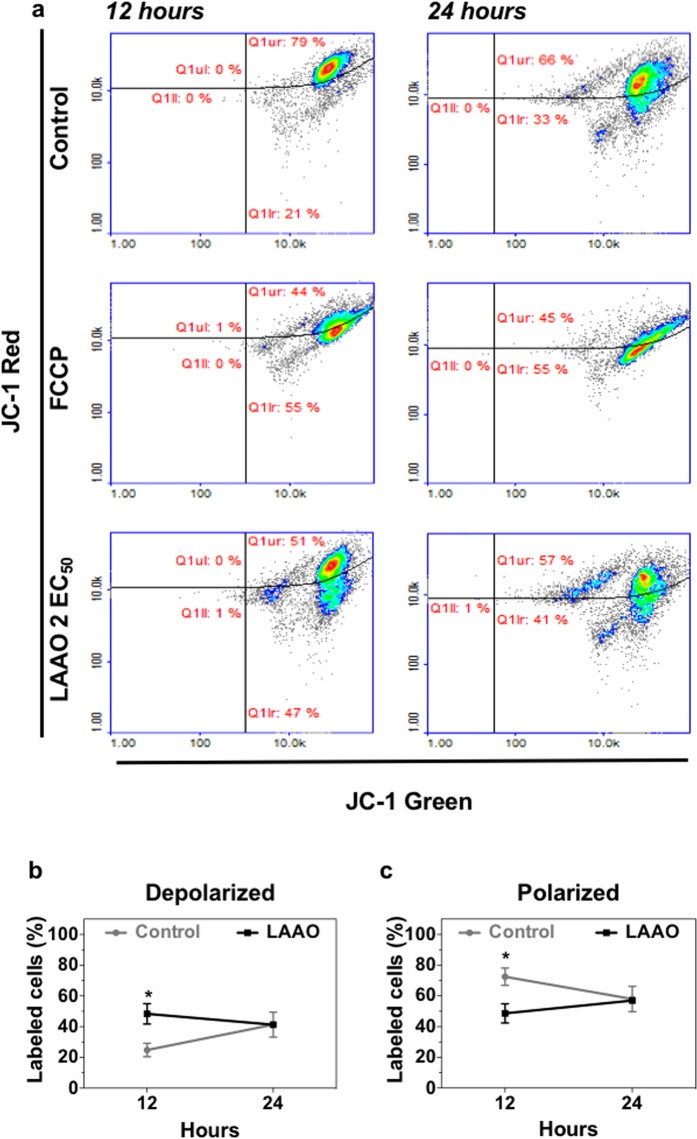
Mitochondrial membrane potential (12 e 24 hours) after LAAO treatment. Keratinocytes were treated with LAAO (2EC_50_) of different time points. **(a)** Dot plots show cells labeled with JC-1 reagent. Non-treated cells were used as negative control and cells treated with 50 μM FCCP were used as positive control. (**b**,**c**) Graphs show quantifications of the proportion of **(b)** Depolarized mitochondrial membrane. **(c)** Polarized mitochondrial membrane. Values represent means of three independent assays and error bars indicate standard error of the means (SEM). *p ≤ 0.05.

To define whether LAAO treatment triggers apoptosis via the intrinsic pathway, the mitochondrial membrane potential was analyzed (Fig. 7). In the intrinsic apoptotic pathway, a reduction of mitochondrial membrane potential results in membrane permeability and culminates in the release of pro-apoptotic proteins^39,44^. After 12 hours treatment with LAAO, a significant increase in the number of cells with depolarized mitochondrial membrane (48% of cells), is observed compared to control (24% of cells) (p ≤ 0.05). Conversely, a reduction was detected in cell mitochondrial membrane polarization after 12 hours, from 72% in control cells to 48% in LAAO-treated cells (p ≤ 0.05). These changes were transient and values returned to control levels by 24 hours incubation.

**Figure 8:**
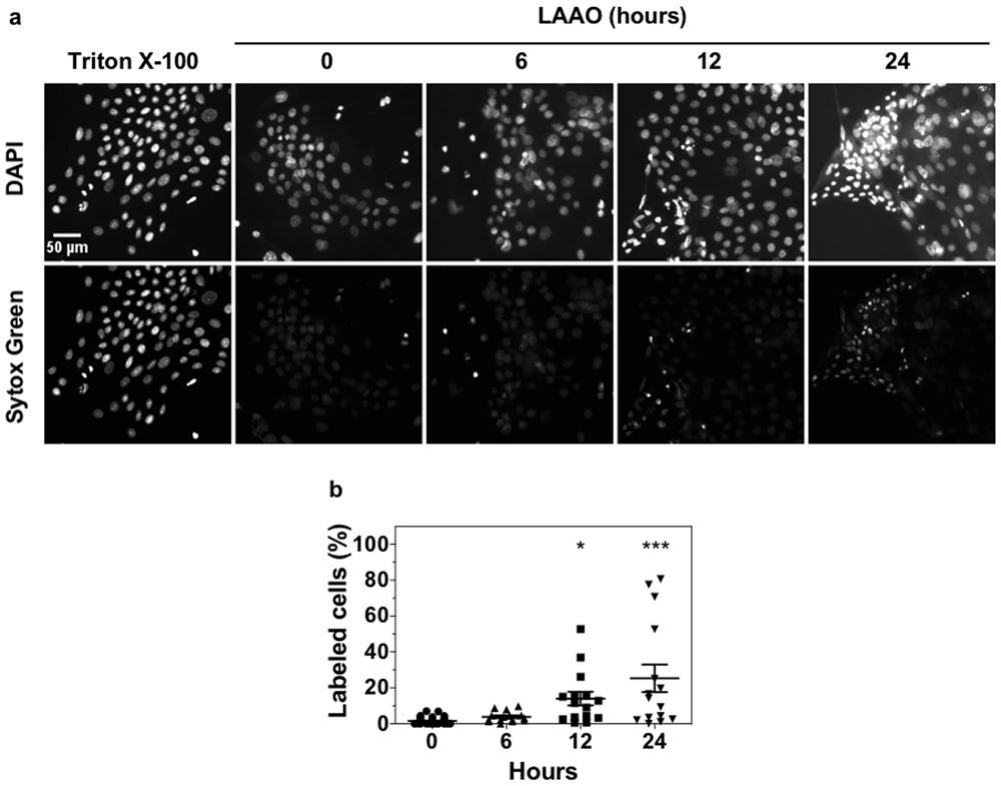
LAAO treatment induces necrosis from 12 hours onwards. Cells were treated with LAAO (2EC_50_) for 6, 12 e 24 h. Cells treated with Triton X-100 0.1% were used as positive control and non-treated cells as negative control. **(a**) Cells were stained with DAPI (nuclei) and Sytox Green. Scale bar = 50 μm. **(b)** Quantification of percentage of necrotic cells using FIJI software. Values represent means of three independent assays and error bars indicate standard error of the means (SEM). *p ≤ 0.05, ***p ≤ 0.001.

The possibility that LAAO could induce cell necrosis was inconclusive when tested by PI staining (Fig. 6e). To confirm whether necrosis is triggered, necrotic cell death was also tested using a more sensitive probe, Sytox Green. This reagent penetrates compromised membranes and exhibit a 500-fold fluorescence enhancement upon binding nucleic acids (as described by the manufacturer). Representative images of cells labeled with DAPI and Sytox Green during a time course are shown (Fig. 8a). An increase in the percentage of necrotic cells identified by Sytox Green labeling was significantly higher after 12 (p ≤ 0.05) and 24 hours (p ≤ 0.001) of LAAO treatment (Fig. 8b). This method was more efficient to detect necrosis than PI, which may be due to the Sytox Green fluorescence enhancement described above. Taken together, we concluded that the decrease in cell viability triggered by LAAO is achieved by distinct cell death mechanisms that are separated temporally.

### LAAO is internalized by keratinocytes

**Figure 9:**
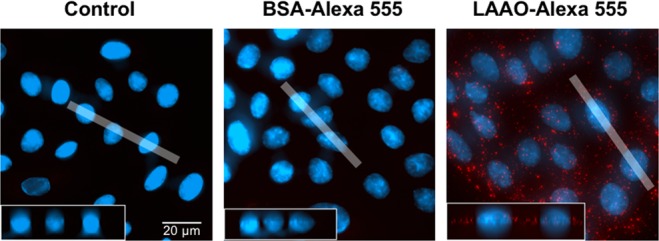
LAAO internalization by keratinocytes. LAAO was labeled with Alexa-555 as manufactor’s instructions and incubated with cells for 1.5 hours. The same procedure was performed for BSA, used control. Untreated cells were used as negative control. Z-profile of 20 pixels wide (white shadow) is projected in Z (shows the height of the cells). Images were acquired in a widefield microscopy. Scale bar = 20 μm.

Once LAAO cytotoxicity was detected, we decided to test whether the enzyme could act intracellularly or would only play its cytotoxic role by interacting with cell membrane (as described previously^45^). For this aim, we investigated whether LAAO would be internalized by cells by labeling the protein with Alexa 555 and incubating with cells. LAAO-Alexa 555 was internalized by keratinocytes after 1.5-hour treatment (Fig. 9). Results show that LAAO is internalized and localized in the cytoplasm, with accumulation at the perinuclear region. This internalization and localization is specific for LAAO as cells incubated with BSA-Alexa 555 did not show any fluorescence signal (negative control).

## Discussion

Dermonecrosis at the snakebite site has been attributed to toxins such as metalloproteases and PLA_2_^11^, while the involvement of LAAOs in tissue destruction has been under appreciated. Here we present evidence that oxidative stress is a key process driving necrotic wounds *in vivo* by *B*. *atrox* venom, and the strong production of H_2_O_2_ via LAAO catalytic reaction may be a contributing factor. *In cellulo* data using normal keratinocytes confirmed our pharmacological inhibition *in vivo* studies and provides potential mechanisms of action of LAAO. LAAO activity contributes to the necrotic symptoms induced by *B*. *atrox* venom in unexpected ways: it triggers the sequential appearance of distinct modes of cell death that collectively cooperate with other snake toxins to drive extensive tissue destruction observed at the bite site.

A previously characterized antioxidant and LAAO inhibitor, NAC^41,42^ diminishes the tissue damage caused by the *B*. *atrox* venom when co-injected in mice. These results are encouraging by suggesting that LAAO-induced oxidative stress may be involved. However, we cannot formally exclude a potential interference of NAC with the function of other enzymes in the venom^46,47^, similar to what described for other inhibitors. Thus, additional experiments need to be performed in order to confirm the precise role of LAAO in the local *in vivo* symptoms. Nevertheless, these results indicate the damaging consequences of LAAO catalysis for a healthy tissue.

LAAO has known cytotoxicity and causes death of tumor cells and pathogens^26,48–51^. While purification of native LAAO has been performed by many laboratories, it is unclear why the potential role of LAAO in dermonecrosis has been overlooked. LAAO purified from *B*. *atrox* snake venom has a molecular weight of approximately 57 kDa, a predicted size of monomers^15,22^, and nine different protein spots are detected when separated in two-dimension electrophoresis (isoelectric point ranging from 5.9 to 6.5). Such pattern indicates the presence of protein isoforms or post-translation modifications, as previously published^14,15,52^. *B*. *atrox* LAAO amino acid sequence (ALL27300.1) and crystal structure have a high degree of conservation across species^53^. LAAO is a thermo-labile enzyme, and is feasible that experimental variability can occur due to loss of LAAO activity from purification or storage^54,55^. We find that appropriate storage^56^ and a pre-activation step prior to each experiment ensure optimal enzyme stability and reproducibility of the cellular defects caused by LAAO.

There is restricted information to explain LAAO toxicity in different tissues *in vivo* and which core mechanisms of cellular homeostasis LAAO can interfere with. At the moment, it is unclear the contribution of LAAO catalysis (*i*.*e*. amino acid oxidation) or ammonia production (by-product of catalysis) to cellular toxicity. We find that impairment of cell viability by exposure to purified LAAO is concentration-dependent and mostly due to H_2_O_2_ action, consistent with previous work^14,17^. Variable and sometimes lower cellular toxicity is observed when *B*. *atrox* LAAO is tested in transformed cell lines (*i*.*e*. higher concentrations are necessary to kill cells)^17,57^. It is highly likely that cell lines (transformed or immortalized) are more resistant to the toxin action, as survival pathways operate that are distinct from those in normal cells. However, data comparison across different studies may be difficult because of: (i) the instability of purified LAAO, as reported herein and (ii) the use of different assays to determine cytotoxicity. Furthermore, the amount of active LAAO used and the end time point investigated may profoundly affect the outcome (see below).

As part of its cytotoxicity program, LAAO induces morphological alterations such as detachment between cells, cell retraction and shrinkage, which may contribute to loss of tissue cohesion and localized tissue damage during envenomation. Our temporal analyses of cell death pathways show that cells treated with LAAO initially undergo autophagy, while apoptosis and necrosis are later events. It is highly likely that, following LAAO treatment, the distinct mechanisms of cell death reported here may be coordinated and cooperate with each other.

To our knowledge, this is the first study to report autophagy in LAAO-treated cells. In contrast to the wealth of information on apoptosis and snake venoms, autophagy is poorly characterized as a response to different toxins. Phospholipase A_2_ (from *B*. *pauloensis*) or crotoxin (from *Crotalus durissus terrificus*) are examples of snake venom’s toxins characterized as autophagy inducers so far^34,58^. However, it is feasible that autophagy may be a general response to distinct toxins present in snake venoms. It is still unknown whether the autophagic process is a cell response to survive the toxic environment or if autophagy is stimulated by LAAO as part of the envenomation process.

An increase in the number of apoptotic cells correlates with pyknotic nuclei, cell retraction and cell rounding in LAAO-treated cells. The transient appearance of mitochondrial membrane depolarization in keratinocytes indicates the involvement of the intrinsic apoptotic pathway, which has been reported for LAAO from *B*. *atrox* and *Agkistrodon acutus* snake venom (ACTX-8) in different cell lines^17,59^. We think it is unlikely that the extrinsic apoptotic pathway is involved, as there is no evidence for a ligand for cell death receptors in bothropic snake venoms^25,60^. It is however feasible that the extrinsic pathway could be activated in a paracrine manner, rather than due to direct action of LAAO.

Finally, it is still unclear whether LAAO acts outside the cellular space or is internalized to cause maximum damage. A direct interaction with the cell exterior is thought to contribute to its toxicity, as LAAO from different snake species appear to bind to bacterial surface in a glycan-dependent manner^61^. However, similar association with the surface of mammalian cells has been controversial^62,63^. In human keratinocytes, a specific internalization of LAAO-Alexa 555 occurs very fast by 1.5 hours, and localize in discrete dots in the cytoplasmic region and around the nucleus. LAAO detection correlate temporally with autophagy and may contribute to cell death mechanisms and structural alterations described in the present work. This pattern is distinct from previous work, where fluorescently labelled LAAO is found inside the nucleus and dispersed in the cytoplasm after 24 hours incubation^64^. The apparent discrepancy in localization may reflect distinct time points used: by 24 hours, apoptosis is already ongoing with alterations in cellular permeability.

In conclusion, we show a previously unappreciated function of LAAO in morphological changes and cellular dysfunction that have a potential impact for dermonecrosis at the snake bite *in vivo*. While our *in cellulo* studies still require to be further validated in other models, our data strongly indicate that LAAO may contribute with the known effects of metalloproteases and phospholipases towards severe tissue disruption. Importantly, LAAO operate from inside cells and its mechanisms of action are complex, with distinct cell death pathways temporally coordinated to reduce cell viability and tissue damage. In future studies, determining the specific molecular regulation among the sequential appearance of cell death mechanisms will highlight potential molecular targets to prevent dermonecrosis in patients.

## Materials and Methods

### Venom and animals

*Bothrops atrox* venom was provided by “Oswaldo Meneses” Serpentarium, Universidad Nacional Mayor de San Marcos from Lima, Peru. Venom was diluted in Milli-Q water and stored at −80 °C until use. Protein concentration was measured by Lowry^65^ or BCA method.

Female Swiss mice (18–22 g) were maintained at the animal facilities of Instituto de Ciências Biológicas of Universidade Federal de Minas Gerais (UFMG), Brazil. Animals received water and food *ad libitum*, under controlled environmental conditions. Experimental protocol was approved by the Ethics Committee in Animal Experimentation of UFMG. All animals received humane care in compliance with the “Guide for the Care and Use of Laboratory Animals” of the National Council for Controlling Animal Experimentation, Ministry of Science, Technology and Innovation (CONCEA/MCTI), Brazil.

### *In vivo* assay

LAAO contribution for the development *B*. *atrox* venom *in vivo* symptom was evaluated in Swiss mice. Animals were divided in four groups of five mice each (1) PBS (negative control), (2) *B*. *atrox* venom (150 µg/animal), (3) *B*. *atrox* venom (150 µg/animal) + NAC (1:50 w/w) or (4) NAC. Mice gastrocnemius muscle was injected with 100 μL of each sample, diluted in PBS. After 72 hours animals were euthanized and the tissue injury was evaluated.

### LAAO purification

The two first chromatographic steps (molecular exclusion and ion exchange) were performed as described by Naumann, *et al*.^14^. Briefly, for molecular exclusion chromatography *B*. *atrox* venom (approx. 2 g) was applied to two 2.5 × 100 cm columns in series packed with Sephacryl S-200, previously equilibrated with ammonium acetate buffer. Elution was carried out using the same buffer at a flow rate of 7 mL/h. Fractions were monitored spectrophotometry at 280 nm and assayed for LAAO activity. Samples with the enzymatic activity were pooled and concentrated using 10 kDa membrane Vivaspin tubes (Sartorious). For ion exchange chromatography, the fraction presenting LAAO activity (approx. 150 mg) was applied to a DEAE Sepharose CL 6B (1.5 × 20 cm) column equilibrated with 50 mM Tris buffer pH 8.5. Proteins were eluted with a 0–0.3 M NaCl linear gradient in the same buffer at a flow rate of 12 mL/h and fractions were monitored at 280 nm, tested for LAAO activity and concentrated using 10 kDa membrane Vivaspin tubes. The selected fraction was then applied to a HiTrap Heparin Hp (1.6 × 2.5 cm) (GE Healthcare, Pittsburg, California,USA) as described by Ciscotto, *et al*.^26^. The column was previously equilibrated with 1 mM Tris buffer pH 6.0 containing 1mM benzamidine and bound proteins were eluted with a 0–1 M NaCl linear gradient in the same buffer at a flow rate of 1 mL/min. The chromatography was performed in HPLC Shimadzu and fractions were monitored at 280 nm. The obtained LAAO was then lyophilized and stored at −80 °C until use.

### Electrophoresis

Protein purity was assessed by 1D and 2D SDS-PAGE (1-DE and 2-DE, respectively) (12%) following Laemmli method^66^. For 2D-electrophoresis, a 7 cm IPG strip with pH between 4–7 (GE Healthcare, Pittsburg, California,USA) was rehydrated with 30 μg of LAAO diluted in 125 μL of a solution containing 0.5% of IPG buffer pH 4–7, 50 mM DTT, 1% of protease inhibitor and rehydration solution (GE Healthcare, Pittsburg, California,USA). The first dimension was performed in Ettan TM IPGphor TM 3 (GE Healthcare, Pittsburg, California,USA) isoelectric focusing unit as described by the manufacturer. The strip was isofocalized through a 5-phase electrophoresis program: 100 V for 1 h, 300 V to reach 200 V/h, 1000 V to reach 300 V/h, 5000 V to reach 4000 V/h, 5000 V to reach 1250 V/h. Prior to the second dimension, proteins were reduced and alkylated by an equilibration buffer (0.04 M Tris-HCl, pH 6.8, 1% SDS; 30% glycerol); containing 4 mg/mL DTT in equilibration buffer and then a 40 mg/mL solution of iodoacetamide. Proteins were visualized with Coomassie Blue staining (0.25% Coomassie Brilliant Blue R-250, 45% methanol and 10% glacial acetic acid).

### L-amino acid oxidase activity, activation and stability analysis

Enzymatic assay for LAAO activity was conducted as described by Bregge-Silva *et al*.^18^, with slight modifications. *B*. *atrox* venom fractions or purified LAAO (2 μg) were incubated in 100 mM Tris-HCl buffer, pH 8.5, 5 mM L-leucine as substrate, horseradish peroxidase (5 U/mL) and 2 mM ortho phenylenediamine (as substrate for peroxidase) for 1 h at 37 °C. The reaction was stopped by adding 50 μL of 2 M H_2_SO_4_ and absorbance was determined at 490 nm using a microplate reader (Bio-Rad - model 680, Hercules, California, USA). The specific activity was expressed as ΔA492 nm/min relative to protein concentration (mg).

LAAO activation was performed by protein incubation with 50 mM sodium acetate buffer pH 5.0 for 30 minutes at 37 °C. For stability assay, two aliquots of LAAO were kept for at least 5 days at 4, −20 or −80 °C. Followed the 5 days, one aliquot at each temperature was activated and the other was tested with no activation.

### Cell Culture

Normal human keratinocytes from neonatal foreskin (strain Sf, passages 3 to 6) are from private collection isolated in 1995 and frozen down. In addition, when necessary, primary cultures of keratinocytes were bought commercially (Lonza). All methods were carried out in accordance with relevant guidelines and regulations. Keratinocytes were cultured on a mitomycin C (Sigma)-treated monolayer of 3T3 fibroblasts at 37 °C and 5% CO_2_ in FAD medium containing 10% of fetal calf serum (FCS), as described previously by Braga *et al*.^67^. Cells were cultured to 60–80% confluence before being used in experiments. For cytotoxicity assay 2.2–3.2 × 10^3^ cells/well were plated in a 96 well microtiter plate. Cells were washed with Versene (PBS containing 0.53 mM EDTA) and pre-incubated with FAD medium containing 1% FCS for 6 hours. In order to analyze cell morphology, necrosis (Sytox Green reagent), autophagy and for LAAO internalization assays, keratinocytes were plated on 13 mm diameter coverslips (2–3 × 10^4^ cells/coverslip). For apoptosis/necrosis and mitochondrial membrane potential assay, keratinocytes were cultured in 60 mm plates (1–2 × 10^5^ cells/plate).

### Cell viability and morphological alterations

LAAO cytotoxicity against keratinocytes was tested using Alamar Blue reagent according to Damico *et al*.^68^, with modifications. Cells were treated with different concentrations (0.625 to 40 μg/mL) of LAAO diluted in FAD medium containing 1% FCS and incubated for 24 hours at 37 °C and 5% CO_2_. Medium containing LAAO was replaced by Alamar Blue 10% (diluted in DMEM without phenol red and containing 1% FCS). The fluorescence was determined after 3 h at 560 nm of excitation and 590 nm of emission in a POLARstar Galaxy fluorimeter using FLUOstar Galaxy software (BMG LABTECH, Ortenberg, Germany). Cell viability was calculated considering values of the mean fluorescence of the control (untreated cells) as 100% of viability. The effective concentration able to reduce by 50% (EC_50_) cell viability was determined from the dose-response curve, using the GraphPad Prism 5 software. To analyze the role of H_2_O_2_, keratinocytes were incubated with different concentrations of LAAO (0–2EC_50_) with or without 100 μg/mL of catalase, a H_2_O_2_ scavenger. Fluorescence values of untreated cells were considered 100% of cell viability.

For morphological studies, cells were treated in the same conditions described for cytotoxicity assay and fixed. Alternatively, keratinocytes were treated with LAAO at 2EC_50_, observed for 6 hours and real-time phase contrast images were taken. Untreated cells were used as control.

### Staining and Microscopy

Keratinocytes were fixed with 3% paraformaldehyde for 10 minutes. When nuclei staining was required, cells were incubated with DAPI (4′,6-diamidino-2-phenylindole) for 15 min and cells were mounted in glass slides using Mowiol. Images from necrosis and autophagy assays were acquired in Olympus Provis BX51 microscope coupled to monochromatic camera SPOT RT using SimplePCI 6 software (Hamamatsu, Japan). For cell morphology, phase contrast images were acquired with 10X objective and for video time-lapse acquisition, real time phase contrast images were acquired every 5 min, during 6 hours with 40X objective in Widefield Zeiss Axio Observer microscope, using Zen acquisition software (Carl Zeiss AG, Oberkochen, Germany). For LAAO internalization analysis, LAAO and Bovine Serum Albumin (BSA) labeling was performed using Alexa Fluor® 555 Microscale Protein Labeling Kit (ThermoFisher Scientific, Leicestershire, United Kingdom) as per manufacturer’s instructions. Images were acquired using 60X objective in Widefield Zeiss Axio Observer microscope, using Zen acquisition software (Carl Zeiss AG, Oberkochen, Germany).

### Apoptosis and Necrosis

Cells were treated with LAAO (2EC_50_) for different time points (6, 12 and 24 h). Treatment with staurosporine (1 μM) or Triton X-100 (0.1%) was used as positive controls for apoptosis and necrosis, respectively. LAAO and positive controls were diluted in FAD medium (1% FCS) and cell incubated with this medium were used as negative control. Cells were trypsinized, centrifuged and resuspended in PBS (approx. 1 × 10^5^ cells). Keratinocytes were centrifuged at 2000 rpm for 2 min and the pellet was resuspended in Annexin V binding buffer (0.1 M Hepes pH 7.4, containing 1.4 mM NaCl and 25 mM CaCl_2_). Annexin V-FITC (1:500) together with Hoechst 3334 (10 μg/mL), were added to the mixture and cells were incubated for 15 min at 37 °C in the dark. Cells were centrifuged, washed with Annexin V binding buffer, and the pellet was resuspended in the same buffer containing Propidium Iodide (PI) (5 μg/mL). Cells were analyzed by flow cytometry (NucleoCounter NC-3000 – ChemoMetec, Allerod, Denmark). Fluorescence was determined at 525/30 nm for Annexin V-FITC and 583/26 nm for PI. Necrosis was also quantified using Sytox Green labeling. After washing with Tris-Buffered Saline (TBS) and fixed in PFA 3%, keratinocytes were incubated with Sytox Green (167 nM) in TBS containing FCS 10% for 30 min at room temperature in the dark.

### Autophagy analysis

Cells were transfected with 0.5 μg/coverslip of LC3-GFP (microtubule associated protein 1 light chain 3) construct using Fugene reagent (Promega) as per manufacturer’s instructions. After 24 hours transfection, cells were incubated with LAAO (2 EC_50_) for 1.5, 3 and 6 hours. Keratinocytes incubated with vehicle were used as negative control, while cells starved (incubated with Earle’s Balanced Salt Solution – EBSS- Sigma) for 30 min were used as positive control. Cells were fixed and stained with DAPI as described above.

### Mitochondrial membrane potential analysis

Keratinocytes were incubated with LAAO (2 EC_50_) for 12 and 24 hours. Cells treated with 50 μM FCCP (Carbonyl cyanide 4-(trifluoromethoxy)phenylhydrazone) for 15 min were used as positive control, while cells incubated with the vehicle were used as negative control. After treatment, cells were trypsinized, centrifuged and resuspended in PBS, followed by incubation with JC-1 (Chemometec) (2.5 μg/mL) at 37 °C for 15 min. After two steps of centrifugation (2000 rpm for 2 min), cells were stained with DAPI and analyzed by flow cytometry (NucleoCounter NC-3000 – ChemoMetec, Allerod, Denmark). JC-1 fluorescence was determined at 529 and 590 nm.

### LAAO internalization

Cells were treated with 10.2 μg/mL (corresponding to 2 EC_50_ of LAAO) LAAO-Alexa 555 in FAD medium 1% FCS or with the same amount of BSA-Alexa 555. Cells incubated with medium only were used as negative control.

### Image quantification

For necrosis analysis using Sytox Green, five images were taken randomly with 10X objective and FIJI software was used to quantify the total number of cells (stained with DAPI) and the number of necrotic cells (stained with Sytox Green). Nuclei identification was accessed by the software functions: “Threshold” > “Fill holes” > “Watershed”; followed by: “Analyze particles” (15–1000 pixels^2^) to quantify the absolute values for each channel (DAPI or Sytox Green). The number of cells stained with Sytox Green was divided by the number of cells stained with DAPI in order to calculate the percentage of necrotic cells.

LC3 puncta was quantified in transfected cells using FIJI software. Images were submitted to the software functions: “Find edges” followed by “Threshold” in order to identify all LC3 puncta per image, excluding non-cellular structures. LC3 puncta was then quantified using “Analyze particles” (0.8–2 pixels^2^), the number of LC3 puncta (per image) was divided by the total number of transfected cells per image, in order to obtain the average of LC3 puncta per cell. Results were then normalized considering control cells (time 0) as 1.

### Statistics

Data were expressed as mean ± standard error of the mean (SEM) of three independent experiments. Statistical analysis was performed using one or two-way ANOVA and Bonferroni post-test in GraphPad Prism software.

## Supplementary information

Supplementary Information

Video from control cells.

Video from cells treated with LAAO.

## Data Availability

All data generated or analyzed during this study are included in this published article (and its Supplementary Information files).
