# Supplementary material for: L-amino acid oxidase from Bothrops atrox snake venom triggers autophagy, apoptosis and necrosis in normal human keratinocytes
Source: Sci Rep. 2019 Jan 28;9:781. doi: 10.1038/s41598-018-37435-4 (PMC6349910; doi:10.1038/s41598-018-37435-4)
Supplement: Supplementary file 1 — Supplementary Information [file 41598_2018_37435_MOESM1_ESM.pdf]

L-amino acid oxidase from *Bothrops atrox* snake venom triggers autophagy, apoptosis and necrosis in normal human keratinocytes.

Fernanda Costal-Oliveira; Stephanie Stransky; Clara Guerra-Duarte; Dayane L. Naves de Souza[a]; Dan E. Vivas-Ruiz; Armando Yarlequé; Eladio Flores Sanchez; Carlos Chávez-Olórtegui; Vania M. M. Braga

SUPPLEMENTARY MATERIAL

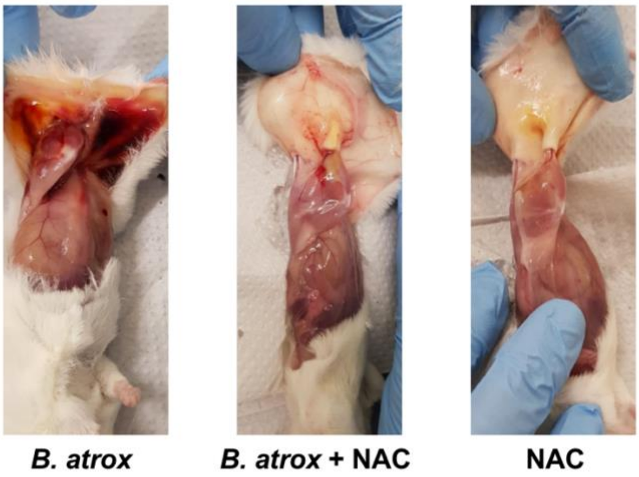

Figure S1: Hemorrhage inhibition by NAC. Mice gastrocnemius muscle was injected with 100 μL of *B. atrox* venom (150 μg/animal), *B. atrox* venom (150 μg/animal) + NAC (1:50 w/w) or NAC. After 72 h animals were euthanized hemorrhage was evaluated.

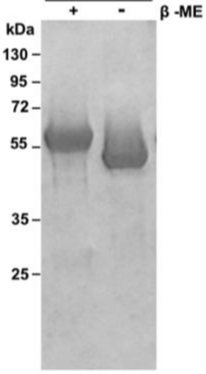

Figure S2: LAAO SDS-PAGE. LAAO with (+) and without (-) β-mercaptoethanol (β-met) was applied to a 12% polyacrylamide gel and protein was stained with Coomassie Blue.

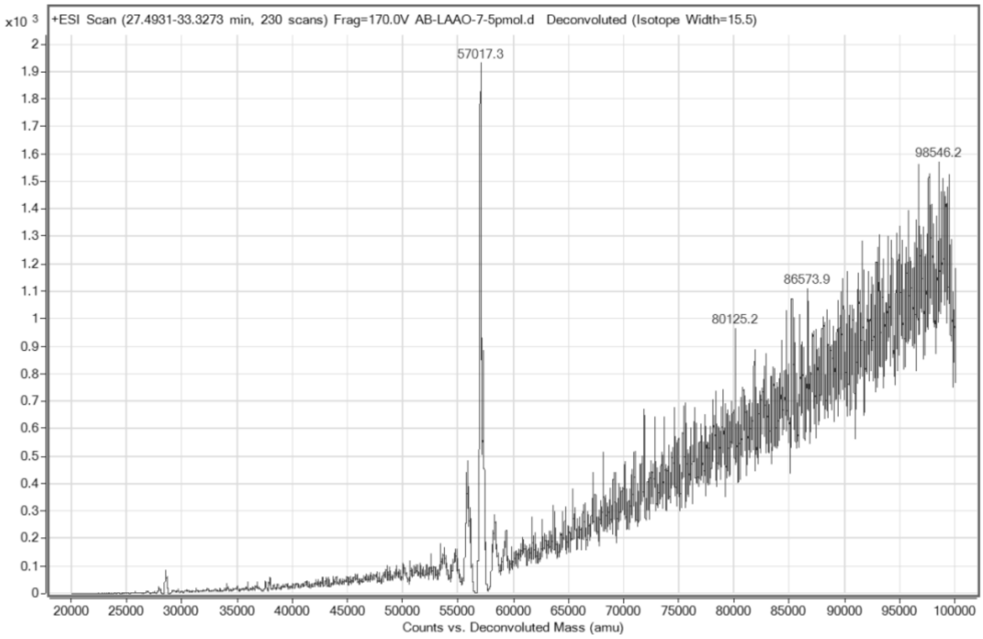

Figure S3: Mass spectrometry analysis of LAAO. LAAO purity was confirmed by mass spectrometry analysis. Protein was commercially analyzed by FingerPrints Proteomics (Dundee – UK).

**Step**	**Protein**		**Specific Activity (U/mg)**	**Purification Factor**
	**mg**	**%**		
***B. atrox* Venom**	8 150	100	6.9	1
**Molecular Exclusion**	981.6	12	9.25	1.35
**Ion Exchange**	73.3	0.9	19.7	2.85
**Heparin Affinity**	23.3	0.28	25.45	3.7

Table S1: Purification of LAAO from *B. atrox* venom.

Table S1 shows that, from 8g of *B. atrox* venom, it was recovered 23mg of purified LAAO (0.28% of the venom). An increase in the specific activity through the purification steps was observed and the final purification factor was 3.7.

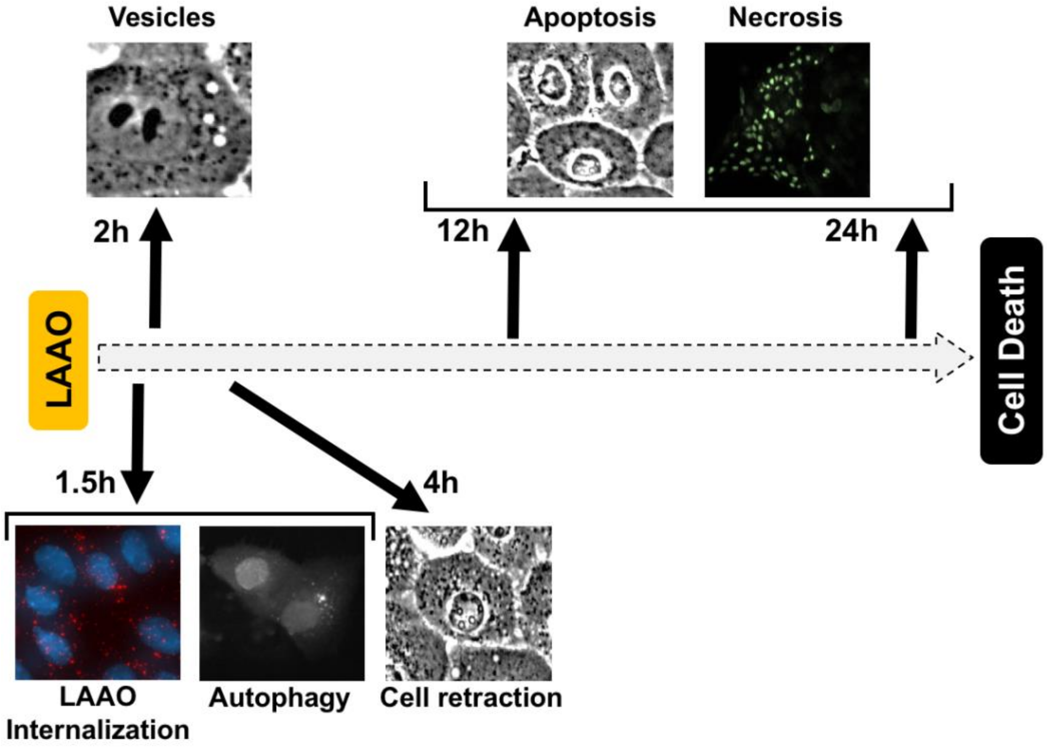

Figure S4: Schematic summary of events triggered by LAAO treatment in keratinocytes.

Video S1: Video from control cells. Non-treated keratinocytes were imaged using phase contrast microscopy for 6 hours. Images were acquired in a widefield time-lapse microscopy. Scale bar=50μm.

Video S2: Video from cells treated with LAAO. Keratinocytes were treated with LAAO ($2EC_{50}$) and imaged using phase contrast microscopy for 6 hours. Images were acquired in a widefield time-lapse microscopy. Scale bar=50μm.
